# Putrescine biosynthesis and export genes are essential for normal growth of avian pathogenic *Escherichia coli*

**DOI:** 10.1186/s12866-018-1355-9

**Published:** 2018-12-27

**Authors:** Priscila R. Guerra, Ana Herrero-Fresno, Victor Ladero, Begoña Redruello, Teresa Pires dos Santos, Malene R. Spiegelhauer, Lotte Jelsbak, John Elmerdahl Olsen

**Affiliations:** 1 Department of Veterinary and Animal Sciences, University of Copenhagen, Stigbøjlen 4, 1870 Frederiksberg C, Denmark; 2 Instituto de Productos Lácteos de Asturias (IPLA-CSIC), Villaviciosa, Spain; 3 Department of Science and Environment, Roskilde University, Roskilde, Denmark

**Keywords:** Fitness, Virulence, *E. coli*, APEC, Polyamines, UHPLC, Gene expression, Membrane stress

## Abstract

**Background:**

Avian pathogenic *Escherichia coli* (APEC) is the infectious agent of a wide variety of avian diseases, which causes substantial economic losses to the poultry industry worldwide. Polyamines contribute to the optimal synthesis of nucleic acids and proteins in bacteria. The objectives of this study were to investigate; i) whether APEC *E. coli* encodes the same systems for biosynthesis and uptake as described for *E. coli* K12 and ii) the role of polyamines during in vitro growth of an avian pathogenic *E. coli* strain (WT-ST117- O83:H4T).

**Results:**

Following whole genome sequencing, polyamine biosynthesis and export genes present in *E. coli* MG1655 (K-12) were found to be identical in WT-ST117. Defined mutants were constructed in putrescine and spermidine biosynthesis pathways (Δ*speB*, Δ*speC*, Δ*speF*, Δ*speB/C* and Δ*speD/E*), and in polyamines transport systems (Δ*potE*, Δ*yeeF*, Δ*potABCD* and Δ*potFGHI*). Contrary to what was observed for MG1655, the Δ*potE*-ST117 mutant was growth attenuated, regardless of putrescine supplementation. The addition of spermidine or orthinine restored the growth to the level of WT-ST117. Growth attenuation after induction of membrane stress by SDS suggested that PotE is involved in protection against this stress. The Δ*speB/C*-ST117 mutant was also growth attenuated in minimal medium. The addition of putrescine or spermidine to the media restored growth rate to the wild type level. The remaining biosynthesis and transport mutants showed a growth similar to that of WT-ST117. Analysis by Ultra-High Performance Liquid Chromatography revealed that the Δ*speB/C* mutant was putrescine-deficient, despite that the gene *speF*, which is also involved in the synthesis of putrescine, was expressed.

**Conclusions:**

Deletion of the putrescine transport system, PotE, or the putrescine biosynthesis pathway genes *speB/C* affected in vitro growth of APEC (ST117- O83:H4) strain, but not *E. coli* MG1655, despite the high similarity of the genetic make-up of biosynthesis and transport genes. Therefore, blocking these metabolic reactions may be a suitable way to prevent APEC growth in the host without disturbing the commensal *E. coli* population.

**Electronic supplementary material:**

The online version of this article (10.1186/s12866-018-1355-9) contains supplementary material, which is available to authorized users.

## Background

Avian pathogenic (APEC) *Escherichia coli* strains belong to the group of extraintestinal pathogenic *E. coli* (ExPEC) [1]. They are the cause of avian colibacillosis [2], which is characterized by multiple organ lesions as airsacculitis, polyserositis, and pericarditis [3]. In recent years, a particular APEC strain has been associated with severe disease outbreaks in the Danish broiler production [4]. This strain, *E. coli* 083:H4 is assigned to the sequence type ST117 and the phylogenetic group D [5].

Polyamines, including putrescine, spermidine, spermine, and cadaverine, are cationic molecules derived from amino acids. Polyamines play an important role in the cell metabolism [6]. They can be involved in bacterial cell-cell signalling [7], motility [8], and are necessary for proper cell division [9]. They also contribute to the optimal synthesis of nucleic acids and proteins used in different growth processes [10]. Studies performed in *Salmonella* [11, 12], *Legionella* [13], *Francisella* [14] and *Shigella* [15] have demonstrated that polyamines are essential for virulence of these intracellular pathogens. Due to this variety of effects, polyamine metabolic pathways may represent a potential novel target for antibiotics [16, 17].

**Fig. 1:**
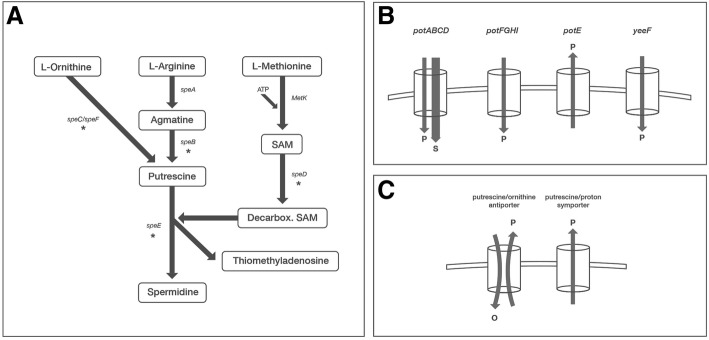
Graphic representation of putrescine/spermidine biosynthesis pathways and transport systems **a**) putrescine/spermidine biosynthesis, where the deleted genes in the present study are indicated by an asterisk; **b**) putrescine/spermidine main transport systems, where ‘p’ represents putrescine and ‘s’ represents spermidine **c**) graphic representation of putrescine/proton symporter and putrescine/ornithine antiporter, where ‘p’ represents putrescine and ‘o’ represents ornithine. The figure is revised from Shah (20) and BioCyc (https://biocyc.org); SAM: S-adenosylmethionine; Decarbox. SAM: Decarboxylated SAM

Multiple putrescine uptakes and degradation systems have been described in *E. coli* K-12, although some associated functions still remain to be completely understood. Intracellular polyamines amounts are regulated by biosynthesis, degradation, excretion, and uptake from the surrounding environment [18]. Putrescine is synthesized from L-arginine by the reactions which are catalysed by the enzymes arginine decarboxylase and agmatinase enzymes. These enzymes are encoded by the genes *speA* and *speB.* Putrescine can also be synthesized from L-ornithine through the action of the two ornithine-decarboxylation enzymes (OCDs), which are encoded by either *speC* or *speF* (Fig. 1) [19, 20]. The substrates for spermidine biosynthesis are putrescine and decarboxylated S-adenosyl-methionine (SAM) and requires enzymes encoded by *speD* and *speE*. Regulation of these biosynthesis processes is not fully understood; however, the intracellular amount of spermidine seems to be self-regulated [21].

The two major recognized systems for polyamine uptake in *E. coli* K12 belong to the family of ATP-binding-cassette (ABC) transporters. They are classified as the spermidine-preferential uptake system (PotABCD) and the putrescine-specific uptake system (PotFGHI) [21]. PotE is a membrane protein involved in the uptake of putrescine via proton symport mechanism, and it has been found to catalyze putrescine efflux by putrescine-ornithine antiport activity in *E. coli* K12 (Fig. 1), [22]. Another putrescine importer system, referred to as YeeF, has also been described. This system depends on proton motive forces [19].

The precise role of polyamine biosynthesis pathways and transport systems has not been studied in pathogenic *E. coli.* Considering that APEC strains have the ability to spread to different body sites, where intestinal *E. coli* do not grow, we hypothesized that polyamine biosynthesis and uptake might be essential for growth of APEC. In order to investigate whether the system described for *E. coli* K12 is present in APEC strains, we first performed a genomic investigation of an ST117 APEC strain. Secondly, we investigated the growth ability of polyamine biosynthesis and transport mutants of the same APEC strain (ST117- O83:H4). As a control, we compared the observed results to those obtained for a standard K-12 strain (MG1655).

## Results

### Characterization of the APEC strain WT-ST117 based on whole genome sequencing (WGS)

The APEC strain WT-ST117 has previously been characterized by phenotypic tests and traditional MLST typing [5] and its virulence potential has been determined [23, 24]. Before being used to analyse the importance of polyamines during growth, it was characterized by whole genome sequencing. This confirmed that the strain belongs to ST-type ST117 and serotype O83:H4 as previously described [5]. It was found to contain several virulence factors, many of which are associated with pathogenicity islands (PAI), such as *papA*, *fimH*, *papEF*, and *ireA,* as well as genes harbored by a large avian *E. coli* plasmid termed pAPEC-O2-ColV (*iroN, sitA, iss)* [24]*.* The gene *feoB,* which has been described in almost all APEC isolates [23], was also found. Other virulence genes identified are also commonly seen among APEC isolates, such as *iucD* and *tsh* (related to pAPEC-O2-ColV), as well as *papC* and *ast,* both associated to adhesin formation [24]*.* Furthermore, the strain was found to carry multiple iron acquisition systems, which may reflect the ability of the bacteria to grow in environments with low concentrations of iron during the infection, as described in Janßen [25]. The isolate was shown to harbour genes encoding resistance to aminoglycosides (*aad*A1) and sulphonamide (*sul*1). This genotype was in accordance with phenotypic resistance results (Additional file 1:Table S2). Besides, the WT-ST117 strain was found to contain pAPEC-O2-ColV (NC_007675) plasmid, and IncFIB/IncFII plasmid replicons.

The polyamine biosynthesis and transport genes described in MG1655 (https://biocyc.org) were conserved and all of them were present in WT-ST117. A summary of the genomic features is given in Table 1. Interestingly, WGS revealed that in the APEC strain, a small sequence encoding 51-amino acid residue of a hypothetical protein (HP), which is not present in the genome of MG1655 in the same position (NC_000913.3), was located immediately upstream of *potE,* as shown in (Additional file 1: Figure S1 )*.* This observation suggests that residues of a particular hypothetical protein may be inserted into different regions [26]. NCBI analysis (https://blast.ncbi.nlm.nih.gov/Blast.cgi#alnHdr_1379240753) and randomly selected isolates on EnteroBase (http://enterobase.warwick.ac.uk/species/ecoli/search_strains?query=st_search) indicate that this genetic configuration is observed in ExPEC isolates (NC_017659.1/NC_011751.1), but it is not commonly found among APEC isolates.

**Table 1:** Main genomic features of the APEC strain *E. coli* ST117

Genome size	5,028,456 bp			
Number of coding sequences	4840			
Number of contigs (with PEGs)	135			
GC %	50.4%			
R-genes (genotype)	V-genes (genotype)	Replicons	MLST-type	Serotype
*aadA1* (aminoglycoside) *sul1* (sulphonamide)	*iroN, sitA, feoB,ireA,irp-2, iucD,vjj, ipfA* (siderophore) *tsh* (serin protease autotransporter) *iss* (increased serum survival) *hlyF* (haemolysin) *fimH, fliH, csgA,papA, papC and papEF* (flagellar proteins) *fliC, ompT,* PAI *(cft073*) (miscellaneous structures) *pstB* (acid shock) *vac, vat, astA* (toxin-associated) *fyuA, gimB, malI, etsA, etsB* (PAI-associated) *upaG, uvrY* (UPEC genes associated) *frz* (oxygen-restricted)	IncFIB IncFII	ST117	O83:H4

### Analysis of growth in vitro

In order to investigate whether the genes involved in putrescine and spermidine biosynthesis and in transport of polyamines are required for growth in minimal medium, we analysed the lag-phase and the growth rates of biosynthesis and transport mutant strains compared to the corresponding wild type strains. Results showed that none of the MG1655 biosynthesis mutants deviated significantly from the WT strain with regards to length of lag-phase or growth rate. This is confirmation of previously published results in *E. coli* K12 [26, 27] (Additional file 1: Figure S2A and S2B).

**Fig. 2:**
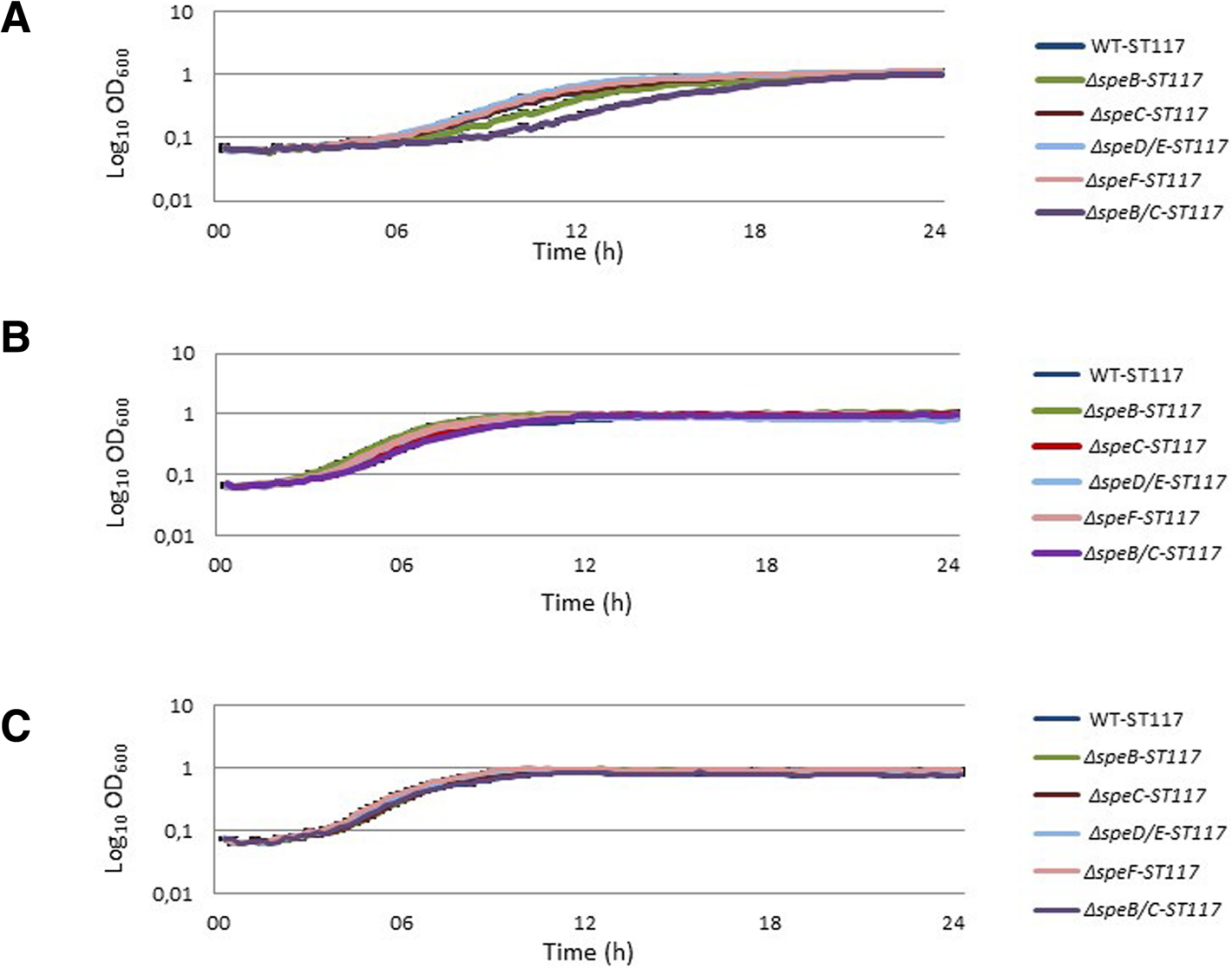
Growth phenotypes of polyamine biosynthesis mutants of *E.coli*-ST117 in **a**) M9-minimal medium; **b**) M9-minimal medium supplemented with putrescine; **c**) M9-minimal medium supplemented with spermidine

A slight increase of the lag-phase was observed for *ΔspeB-*ST117 and its growth rate, although not significant, was below the growth rate of the WT-ST117 in minimal medium, while the mutant in *speC* grew exactly as the wild type strain (Fig. 2a). A significant increase of the lag-phase and a significantly reduced growth rate (μ = 0.27 compared to μ = 0.52 for WT, *p* = 0.009) were observed in the double biosynthesis mutant (Δ*speB/C-*ST117) when grown in minimal medium (Fig. 2a) (Table 2). WGS confirmed that the Δ*speB/C* mutant lacked the expected specific genes, which were replaced with selectable markers (chloramphenicol/trimethoprim), and that no additional mutations were present in the strain. The results were confirmed by basic local alignment search tool (BLAST) (https://blast.ncbi.nlm.nih.gov/Blast.cgi) and alignment software MAUVE (http://darlinglab.org/mauve/mauve.html). Thus, every phenotype observed for the mutant could unambiguously be related to the site-specific mutations.

**Table 2:** Growth rates of selected polyamine biosynthesis and transport system mutants

Growth condition	Growth rate		
	WT-ST117	Δ*speB/C-*ST117	Δ*potE-*ST117
M9 minimal medium	0.52 ± 0.04	0.27 ± 0.02^a**^	0.26 ± 0.00^a**^
M9 + putrescine	0.46 ± 0.02 ^b*^	0.46 ± 0.01 ^b*^	0.37 ± 0.01^a**,b*^
M9 + spermidine	0.42 ± 0.01 ^b**^	0.44 ± 0.06 ^b*^	0.43 ± 0.02 ^b*^
M9 + ornithine	0.41 ± 0.01 ^b*^	0.30 ± 0.01 ^a**^	0.41 ± 0.01 ^b*^
M9+ arginine	0.47 ± 0.00 ^b*^	0.40 ± 0.01^a*,b*^	0.28 ± 0.06^a**^

^a^growth rates marked with ^a^ are significantly different from the growth rate of WT-ST117 growing at the same condition; mean values ± standard deviation

^b^growth rates marked with ^b^ are significantly different from the growth rate of the same strain growing in M9 minimal medium without supplementation; mean values ± standard deviation; *p < 0.05;** *p* < 0.01

When the media was supplemented with putrescine or spermidine, the growth performance of the mutant was similar to that of the WT-ST117 (Fig. 2b and c), showing that extracellular polyamines could compensate for lack of biosynthesis. Construction of a triple mutant (Δ*speB/C/F-*ST117) was not possible, with any of the techniques used. The remaining biosynthesis mutants grew similarly to WT-ST117 in all the media tested (Fig. 2a, b and c); thus lack of production of spermidine due to knock-out of *speD* together with *speE* was not crucial for the growth of the bacterium, even when growing in M9, which does not contain this polyamine.

**Fig. 3:**
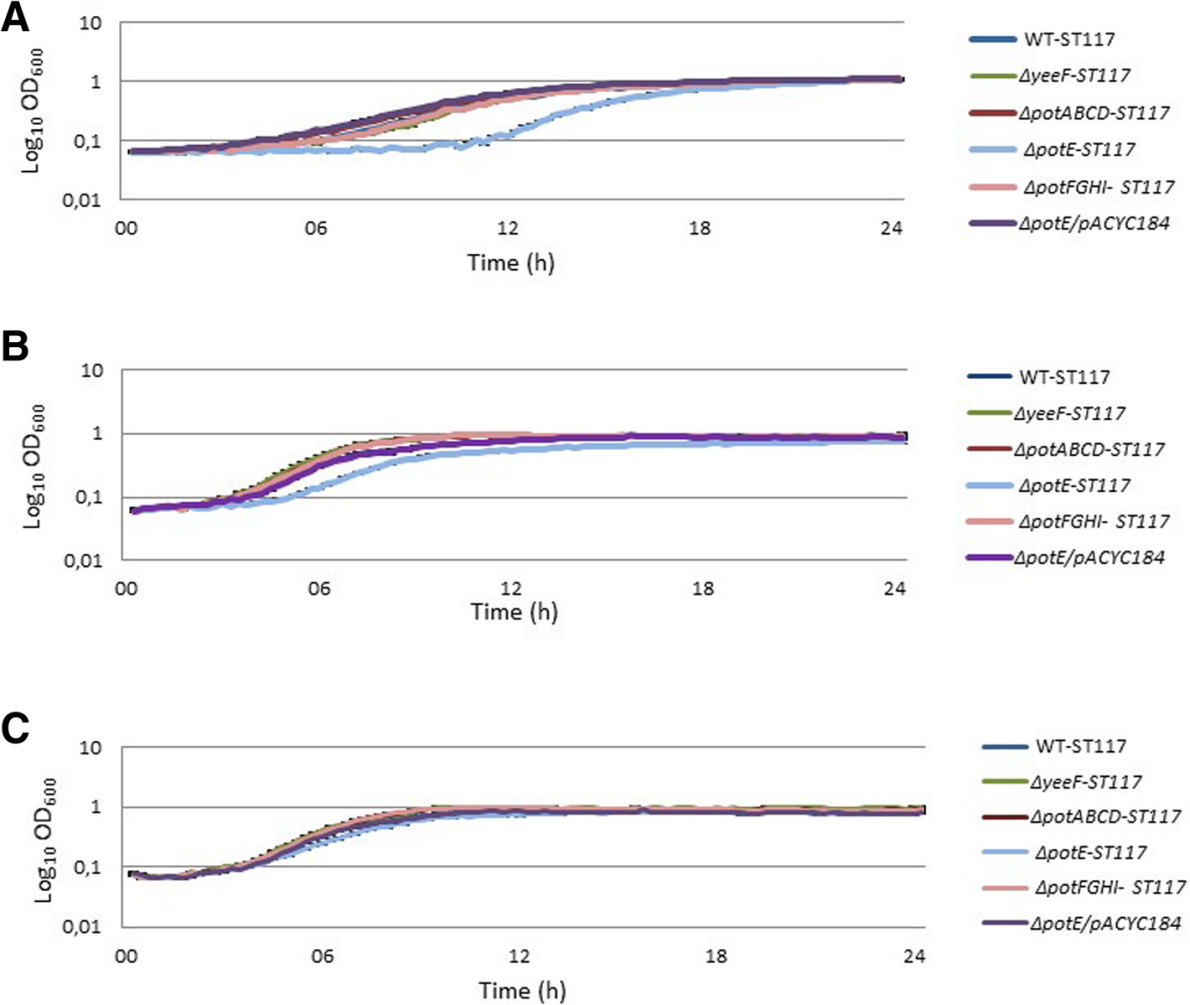
Growth phenotypes of polyamine transport mutants of *E.coli* WT-ST117 in **a**) M9-minimal medium; **b**) M9-minimal medium supplemented with putrescine; **c**) M9-minimal medium supplemented with spermidine

Mutants of MG1655 in polyamine transport-systems grew similar to wild type MG1655 in minimal media whether supplemented with polyamines or not (Additional file 1: Figure S2B). Mutants in polyamine transport systems of ST117 also grew similar to the wild type strain, with the exception of *ΔpotE*-ST117, (Fig. 3a, b and c).

The *ΔpotE*-ST117 mutant showed an increased lag-phase and a significantly reduced growth rate in M9 medium (μ = 0.26 compared to μ = 0.52 for WT, *p* = 0.005) (Table 2), although it could reach the same OD as WT-ST117 after 16 h (Fig. 3a). Addition of exogenous putrescine to the medium shortened the lag-phase of the *ΔpotE*-ST117 mutant; however, the growth rate was still significantly different from the WT-ST117 (μ = 0.37 compared to μ = 0.46 for WT, p = 0.005) (Fig. 3b). Furthermore, it was significantly higher than in medium not supplemented with putrescine (Table 2). Complementation with the *potE* gene *in trans* (Δ*potE*/pACYC184) restored the wild type phenotypes (Fig. 3a and b). Interestingly, when adding spermidine to the minimal medium, *ΔpotE*-ST117 grew similarly to WT-ST117 (Fig. 3c).

**Fig. 4:**
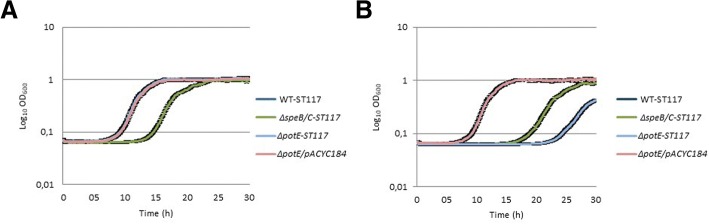
Growth phenotypes of polyamine biosynthesis and transport mutants of *E.coli* WT-ST117 in **a**) M9-minimal medium supplemented with ornithine; **b**) M9-minimal medium supplemented with arginine

We also investigated the growth performance of the WT-ST117, the Δ*speB/C*, *ΔpotE* mutants as well as the Δ*potE*/pACYC184 complemented mutant in minimal medium supplemented with ornithine or arginine, since these two amino acids are putrescine precursors, and ornithine can be exchanged by the PotE antiport mechanism (Fig. 1). The addition of exogenous ornithine and arginine to the media resulted in an increase of the lag-phase of *ΔspeB/C*-ST117, (over 10 h delay), and still, the strain showed a significantly reduced growth rate (μ = 0.30 compared to μ = 0.41 for WT; *p* = 0.002) (Fig. 4a) and (μ = 0.40 compared to μ = 0.47 for WT; *p* = 0.03) (Fig. 4b) (Table 2), respectively. In contrast, the addition of ornithine restored *ΔpotE-*ST117 growth rate to the levels of WT-ST117 (Fig. 4a). The *ΔpotE-*ST117 mutant showed a significantly increased lag-phase and significantly reduced growth rate compared with the WT (μ = 0.28 compared to μ = 0.47 *p* = 0.002), when grown in M9 supplemented with arginine (Fig. 4b) (Table 2). To ensure that none of the growth results were artefacts of the small volume of media used in the Bioscreen format, the growth experiments with ornithine and arginine were repeated in 100 ml media in flasks. This showed that growth curves determined by Bioscreen automated system were in agreement with growth curves determined by the traditional cultivation method (Additional file 1: Figure S3 and Additional file 1: Table S1).

### Analysis of the intracellular amount of putrescine during growth

**Fig. 5:**
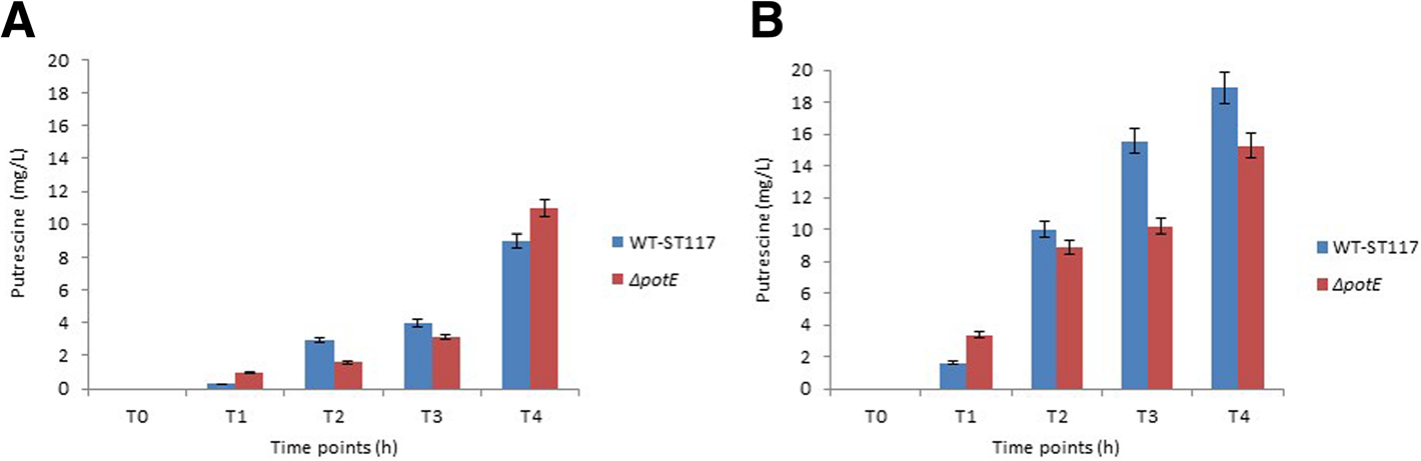
Intracellular putrescine concentration in the strain *E.coli* WT-ST117 and the mutant *ΔpotE*-ST117 in different media **a**) M9-minimal medium; **b**) M9-minimal medium supplemented with putrescine

Since the growth experiments showed that two mutants reported to be involved in intracellular putrescine homeostasis affected the growth rate of the APEC strain under study, we analysed the intracellular putrescine levels in the WT strains and in Δ*speB/C-*ST117 and Δ*potE-*ST117 by UHPLC during growth in minimal medium with or without supplement of putrescine. The analyses revealed that the intracellular amount of putrescine in the Δ*potE-*ST117 mutant was not significantly different from the WT-ST117 strain at any of the time points tested, irrespective of the media used to analyse the growth (Fig. 5). The general trend was the detection of lower values of intracellular putrescine when the bacteria were grown in minimal medium compared with the growth in medium supplemented with putrescine. In M9, until late stationary phase, the Δ*potE-*ST117 mutant showed slightly lower values compared to the WT-STT17 strain, while at late stationary phase, there was a tendency that the mutant accumulated putrescine and reached higher intracellular levels (not significant) than those found in the WT-ST117 strain (Fig. 5a). When exogenous putrescine was added to the medium, the trend was the same, but in this case, the Δ*potE-*ST117 mutant showed lower intracellular levels of putrescine at late stationary phase compared to WT-ST117 (not significant). Besides, in the supplemented medium, both strains showed higher intracellular levels of putrescine at each time point tested than at the equivalent time points analysed in M9, but also these differences were not significant (Fig. 5b).

**Fig. 6:**
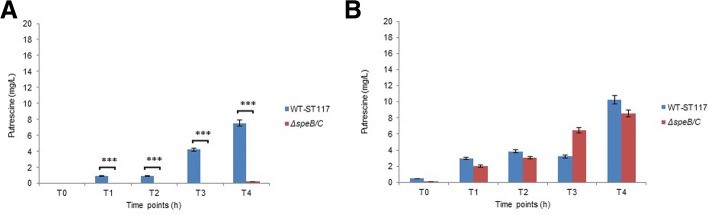
Intracellular putrescine concentration in the strain *E.coli* WT-ST117 and the mutant *ΔspeB/C*-ST117 in different medium **a**) M9-minimal medium; **b**) M9-minimal medium supplemented with putrescine; *** *p* < 0.001

The double biosynthesis mutant, Δ*speB/C-*ST117 had a remarkable lower putrescine intracellular concentration than WT-ST117 in minimal medium. The difference was significant at all-time points tested (Fig. 6a). When exogenous putrescine was added to the medium, the amount of intracellular putrescine was similar and not significantly different to the amount observed inside the WT-ST117 at all-time points (Fig. 6b).

### Analysis of membrane stress caused by SDS

**Fig. 7:**
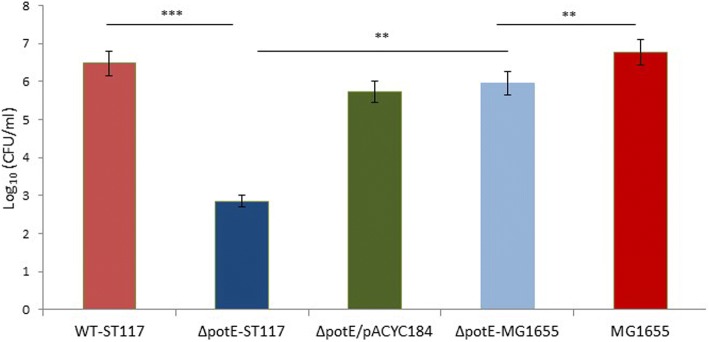
Growth of WT-ST117, Δ*potE*-ST117, and Δ*potE*/pACYC184 strains in the presence of 0.1% (*w*/*v*) SDS; *** *p* < 0.001

In order to investigate whether PotE might have a role in protection against membrane stress in APEC, as it has been described for a range of eukaryotic and prokaryotic organisms [28], we analysed if the mutant lacking *potE* showed increased sensitivity to SDS, which is the prototype stress factor for shock proteins [29]. The assays were performed with WT-ST117 as well as the mutant Δ*potE*-ST117 and Δ*potE*/pACYC184 (complemented) strains. A significantly lower CFU counts was observed for the Δ*potE*-ST117 and Δ*potE*-MG1655 mutants compared with their respective wild type strains when isolates were exposed to a concentration of 0.1% (*w*/*v*) SDS (Fig. 7). The CFU counts of Δ*potE*-ST117 mutant were significantly lower than those observed in Δ*potE*-MG1655 (Fig. 7), suggesting that this mutant was more severely affected by SDS induced stress. The Δ*potE*/pACYC184 strain showed similar CFU counts to those detected for the WT strain as expected (Fig. 7). All the strains grew similarly to the WT when the medium was not supplemented with SDS or supplemented with a concentration of 0.01% (w/v) SDS (Additional file 1: Figure S4).

### Gene expression of polyamine biosynthesis genes

According to the UHPLC results, the double biosynthesis mutant (Δ*speB/C*-ST117) was putrescine-deficient in minimal medium, despite the presence of the *speF* gene. We therefore analysed whether the mutant expressed the *speF* gene, since this gene encodes a redundant enzyme for the same reaction where *speC* is involved in (Fig. 1). In addition, the expression of *speE,* which encodes the enzyme responsible for conversion of putrescine to spermidine (Fig. 1) was analysed. Results showed that both genes were expressed during growth in minimal medium, at all-time points tested (Additional file 1: Figure S5).

## Discussion

In this study, we investigated whether genes involved in biosynthesis or transport of polyamines were required for the in vitro growth of an APEC *E. coli* isolate, WT-ST117, despite the fact that they are dispensable for growth in *E. coli* K12 [26, 27]*.* For this purpose, the growth performance of several mutants was compared with that of WT-ST117. Our hypothesis was that the concomitant blocking of the agmatine and the ornithine pathways would inhibit the synthesis of putrescine and would lead to a significant fitness cost in APEC *E. coli* when the bacteria grow under minimal conditions.

The results showed that the phenotype of a single gene knock-out was stronger (slightly attenuation of growth rate observed) for Δ*speB-*ST117 than for Δ*speC-*ST117, suggesting that *speB* is more important than *speC* for putrescine biosynthesis in APEC WT-ST117 and that arginine might be preferred over ornithine as the source for the biosynthesis. Furthermore, we observed that the double mutant, *ΔspeB/C*-ST117 showed an increased lag-phase and a significant delay of growth under minimal conditions. Addition of putrescine or spermidine to the medium allowed the mutant to grow similarly to the wild type strain, showing that uptake of putrescine from the environment could complement fully for the lack of biosynthesis. Spermidine was apparently not essential for growth since the double mutant without *speD* and *SpeE* grew similarly to the wild type strain. Curiously, the addition of spermidine to the medium compensated for the lack of putrescine biosynthesis. This suggests that spemidine is taken up and then converted by either *speE* or other enzymes to putrescine, in the lack of putrescine biosynthesis. This has not previously been described in bacteria, however, it is well known from eukaryotic polyamine biosynthesis systems that spermidine can be converted to putrescine [30]. On the other hand, conversion of spermidine to putrescine in the absence of an external source of spermidine was not sufficient to compensate the lack of putrescine production, and further studies are warranted to understand the regulation of this system.

Similar study in *Streptococcus pneumoniae* polyamine-deficient mutant pointed that putrescine biosynthesis is crucial for fitness [16]. However, previous studies with *E. coli-*HT306 [26] and similar work carried out on the close relative *Salmonella* [11], demonstrated that polyamines are not required for growth in vitro*,* although polyamines were shown to be essential for systemic infection of mice with *Salmonella* [31]. Thus, despite the close genetic relationship to other strains of *E. coli* and to *Salmonella*, it seems that deletions in the polyamines biosynthesis pathways in the pathogenic APEC *E. coli* WT-ST117 strain is crucial to growth, and it may indicate that polyamines play a different role during APEC (ST117- O83:H4) growth than the described in these other bacteria.

We measured the amount of intracellular putrescine inside WT-ST117 and the isogenic strain *ΔspeB/C*-ST117 at different phases of growth by UHPLC and found that the amount of putrescine within WT-ST117 changed over time, increasing with growth, probably due to the bacterial multiplication. The double biosynthesis mutant was clearly putrescine-deficient and only very low levels of putrescine were detectable at just one-time point. This finding shows that the double biosynthesis mutant does not generate putrescine during growth in minimal medium, or the amounts of synthesized putrescine are not detectable by UHPLC.

Theoretically, the *ΔspeB/C*-ST117 mutant should still have been able to synthesize putrescine from ornithine because the *speF* gene remains present. This was not the case, and we observed that addition of ornithine to the medium, which is the substrate for *speF*, did not restore the growth defect. We cannot fully rule out that the lack of putrescine in this mutant, despite the presence of SpeF, might be due to continuous conversion of putrescine into spermidine catalyzed by SpeE. However, we consider this an unlikely explanation since spermidine appeared to be less important to the cell than putrescine. Nevertheless, under the growth conditions tested, both *speF* and *speE* were expressed in *ΔspeB/C*-ST117.

We also observed that the mutant lacking the gene *ΔpotE*, involved in putrescine homeostasis through transport of putrescine and ornithine, showed an increased lag-phase and significantly reduced the growth rate in minimal medium. It was also growth attenuated in minimal medium supplemented with arginine. The amino acid L-arginine has been described as an inhibitor of bacterial proliferation at certain concentrations due to the effects on bacteria cell membrane leading to disruption and depolarization [32]. This may partly explain the growth arrest in the medium where this amino acid was added. In contrast, the addition of spermidine or ornithine to the media, allowed the mutant to grow similarly to the WT-ST117 strain.

Our first hypothesis to explain the growth attenuation of this mutant was that the lack of the transport system, PotE, might lead to intracellular accumulation of putrescine. Such an explanation would be consistent with the fact that the PotE protein has been described as the main protein associated with the excretion of putrescine [21]. However, our results demonstrated that such intracellular accumulation of putrescine did not take place in any of the media tested; actually, putrescine was found inside of the mutant at lower levels than those observed inside of WT-ST117, even when grown in M9 medium supplemented with putrescine. Therefore, putrescine might either be produced in balanced levels eliminating the need for excretions, or it may be excreted through another transport system or metabolized with other purposes in the mutant. Our findings suggest that the function of PotE may be wider than previously described [22, 34], and since ornithine and spermidine added to the medium eliminated the growth attenuation, it may be an uptake system for these substances; however, it was considered beyond the scope of the current work to investigate the uptake and export repertoire of this molecule.

Reactive oxygen species (ROS) are formed as part of normal cell metabolism. In mammals, oxidation of polyamines can generate highly toxic products such as acrolein, which causes oxidative damage to the cells [30]. In yeast cells, it has been described that central components of the stress response are regulated by polyamine exporter systems [33]. The lack or the excess of polyamines can induce oxidative stress in *E. coli*; the increase of oxidant levels are associated with changes in the metabolism and cell cycle arrests [34, 35]. In light of this, our second hypothesis was that the absence of the transport system (PotE) might lead to a decreased ability to cope with oxidative stress and that the bacterial cells react to the increased oxidative level by arresting the growth. Notably, our findings suggest that PotE has a protective role against oxidative stress in the *E. coli* APEC strain since the mutant Δ*potE-*STT17 was vulnerable to SDS induced membrane stress. Interestedly, the stress oxidase induction seems to be critical in the pathogenic strain. This would also explain with addition of arginine increased the growth attenuation of the Δ*potE* mutants, since this molecule may add to membrane stress [33]. Therefore, the absence of the PotE membrane protein may compromise the pathogenic bacteria survival under stressful conditions.

## Conclusions

Putrescine biosynthesis was found to be important for APEC (ST117- O83:H4) when growing in minimal medium, a phenotype which differs from the phenotype previously described for other types of *E. coli* [26]. Also the PotE transport system was found to be important for growth. One function of polyamines is to maintain the charge balance of the cell and act as a shield stabilizing the cell membrane [36, 37]. Considering that PotE is a putrescine membrane exporter [22], our results indicate that the growth arrest observed for this mutant is a response to oxidative stress. Thus, we suggest that the PotE membrane protein plays a different role during growth of the pathogenic APEC *E. coli* (ST117- O83:H4) strain than during growth of standard laboratory strains, or that these strains have additional systems to cope with membrane stress caused by polyamine imbalance. Despite numerous attempts, we were not able to obtain mutants lacking both *potE* (involved in transport of putrescine) and *speB/C* (required for biosynthesis of putrescine) in our APEC strain (ST117- O83:H4). This suggests that at least one of the systems needs to be intact for any growth to occur.

## Methods

### Bacterial strains and growth conditions

Experiments were performed with an APEC *E. coli* wild type strain O83:H4 (WT-ST117) [5], and *E. coli* MG1655 (K-12) (MG1655) from the strain collection at Department of Veterinary and Animal Sciences, University of Copenhagen. Mutants of these two strains were created as part of the current study (see below). The WT-ST117 was originally isolated from chronic lesions of salpingo-peritonitis in a broiler breeder in Denmark, and belongs to serotype O83:H4, sequence type ST117 and phylogroup D [5]. Prior to growth experiments, bacterial strains were propagated for 16 to 24 h at 30–37 °C in Luria-Bertani (LB) broth (Oxoid) with continuous shaking (200 rpm), or they were cultured overnight on LB agar plates, supplemented with gentamicin 20 μg/ml (Sigma-Aldrich), trimethoprim 10 μg/ml (Sigma-Aldrich) and/or chloramphenicol 5 μg/ml (Sigma-Aldrich) when relevant.

### Mutagenesis

Single gene deletions and concomitant insertion of antibiotic resistance cassettes in WT-ST117 and MG1655 were performed using Lambda Red recombination [38], with the exception of the double mutant ∆*speB/C*, which was obtained by using the In-fusion HD method (Clontech). Genetic complementation of *potE* in WT-ST117 was achieved by cloning the *pot*E gene into the plasmid pACYC184 using standard techniques. The resulting plasmid was electroporated into *E. coli* K-12 (ER2420), from which it was recovered and eventually electroporated into the Δ*potE*-ST117 mutant as described [39]. Mutated strains and plasmids used for mutagenesis are listed in Table 3, while primers used for amplification of the genes are listed in (Additional file 1: Table S3). All constructs were verified by PCR amplification, restriction analysis and sequencing.

**Table 3:** Strains and plasmids used in the study

Strains and plasmids	Relevant features^a,b^	Reference
*E. coli* strains		
*E. coli* (WT-ST117)	Virulent reference strain, APEC strain	[5]
MG1655	MG1655/K-12- Reference strain	ATCC 2592
*E. coli* K-12 ER2420	Cloning intermediate strain	Termo Scientific
Δ*speB*-ST117	*speB* mutant, Chl^R^	This work
Δ*speC-*ST117	*speC* mutant, Chl^R^	This work
Δ*speB/C*-ST117	*speB* mutant*, speC* mutant, Chl^R^, Tmp^R^	This work
Δ*speF*-ST117	*speF* mutant, Chl^R^	This work
Δ*speD/E*-ST117	*speD* mutant, spe*E* mutant, Chl^R^	This work
Δ*potABCD*-ST117	*potABCD* mutant, Chl^R^	This work
Δ*potFGHI*-ST117	*potFGHI* mutant, Chl^R^	This work
Δ*potE*-ST117	*potE* mutant, Chl^R^	This work
Δ*potE*/pACYC184	*potE* mutant, Chl ^R^/ *potE* gene, Tet^R^	This work
Δ*yeeF*-ST117	*yeeF* mutant, Chl^R^	This work
Δ*speB*-MG1655	*speB* mutant, Chl^R^	This work
Δ*speC*-MG1655	*speC* mutant, Chl^R^	This work
Δ*speB/C*-MG1655	*speB* mutant*, speC* mutant, Chl^R^, Tmp^R^	This work
Δ*speF*-MG1655	*speF* mutant*,* Chl^R^	This work
Δ*speD/E*-MG1655	*speD* mutant, *speE* mutant, Chl^R^	This work
Δ*potABCD* -MG1655	*potABCD* mutant, Chl^R^	This work
Δ*potFGHI* -MG1655	*potFGHI* mutant, Chl^R^	This work
Δ*potE* -MG1655	*potE* mutant, Chl^R^	This work
Δ*potF*-MG1655	*potE* mutant, Chl^R^	This work
Plasmids		
pKD46	Plasmid with λ-Red recombinase expressed from arabinose inducible promoter	[39]
pKD3	Template plasmid for λ -red mutagenesis, Chl^R^, Amp^R^	Termo Scientific
pJET 1.2	Cloning vector, Amp^R^	Termo Scientific
pACYC184	Expressing the Δ*potE* gene, Chl^R^, Tet ^R^	Termo Scientific
pM3224T	Template plasmid for Tmp^R^	[44]

^a^The metabolic pathway affected for each mutant is indicated in Fig. 1

^b^Chl^R^ chloramphenicol resistant; Tet^R^ tetracycline resistant; Gen^R^ gentamicin resistant; Tmp^R^ trimethoprim resistant; Amp^R^ ampicillin resistant

### Whole genome sequencing

Genomic DNA was extracted from WT-ST117 and its isogenic ∆*potE-*ST117, Δ*speD/E-*ST117, and ∆*speB/C*-ST117 strains using the Invitrogen Easy-DNATM Kit (Invitrogen) as recommended by the supplier. DNA concentrations were determined by the Qubits DNA BR assay Kit (Invitrogen). The genomic DNA was prepared for Illumina pair-end sequencing using the Illumina (CD genomics) NexteraXT Guide 150,319,425,031,942 following the protocol revision C (https://support.illumina.com/downloads/nextera_xt_sample_preparation_guide_15031942.html). A sample of the pooled NexteraXT Libraries was loaded onto an Illumina MiSeq reagent cartridge by MiSeq Reagent Kit v2 and 500 cycles with a Standard Flow Cell. The libraries were sequenced using an Illumina platform MiSeq v2.6. The isolates were pair-end sequenced. The raw reads were assembled and contigs aligned using CLC Workbench software (CLC Bio-Qiagen). The assembled genome of WT-ST117 and polyamine mutants was submitted to European Nucleotide Archive (ENA; http://www.ebi.ac.uk/ena) under study accession number ‘PRJEB18262’ and the samples accession numbers are ‘ERS1461989’ (WT-ST117), ‘ERS2614665’ (Δ*speD/E-*ST117), ‘ERS2614666’ (∆*speB/C*-ST117), ‘ERS2614667’ (∆*potE-*ST117).

The assembled sequences were analyzed using the pipelines available at the Centre for Genomic Epidemiology (CGE; http://www.genomicepidemiology.org); KmerFinder (version 2; https://cge.cbs.dtu.dk/services/KmerFinder/), and SeroTypeFinder (version 1.1; https://cge.cbs.dtu.dk/services/SerotypeFinder). Other genotypic features such as plasmid replicons, antimicrobial resistance genes, and virulence genes were also identified with a selected threshold equal to 98% identity using the pipelines; PlasmidFinder (version 1.3; https://cge.cbs.dtu.dk/services/PlasmidFinder) ResFinder (version 2.1; https://cge.cbs.dtu.dk/services/ResFinder) and VirulenceFinder (version 1.4; https://cge.cbs.dtu.dk/services/VirulenceFinder) also available at CGE. Specific genes, such as those of the polyamine biosynthesis and transport systems were identified by Blast (https://blast.ncbi.nlm.nih.gov/Blast.cgi) using the sequence of *E.coli* MG1655 (https://www.ncbi.nlm.nih.gov/nuccore/NC_000913.3) as the reference. The sequences of ∆*potE-*ST117 and ∆*speB/C*-STS117 were compared to that of WT-ST117 to confirm that gene knock-outs were site-specific.

### In vitro growth assays

Growth experiments were performed in biological triplicate on BioScreenC (Labsystems) for 24 h at 37 °C. Bacteria were grown overnight in M9-minimal medium (2 mM MgSO_4_, 0.1 mM CaCl_2_, 0.4% glucose, 8.5 mM NaCl, 42 mM Na_2_HPO_4_, 22 mM KH_2_PO_4_, and 18.6 mM NH_4_Cl) and adjusted to the same OD (OD_600_ = 0.05). A volume of 190 μl from each medium tested; M9 and M9 supplemented with putrescine 1.13 mM or spermidine 0.07 mM was added in the honeycomb Bioscreen wells and 10 μl of the bacterial suspension was inoculated. The OD values (recorded with a 600 nm filter) were measured every 15 min with continuous shaking at 37 °C. In addition, the medium was added to several wells and used as blank control. Growth curves were created on the basis of a non-linear model of the log-transformed OD_600_ values with the definition of lag-phase as the time necessary to reach an OD_600_ of 0.1. The maximum specific growth rate (μ) was calculated according to the logistic equation model using the biological triplicate values [40].

In addition, biological triplicate growth assays of WT-ST117, Δ*potE*-T117, Δ*speB/C*-ST117 and Δ*potE*/pACYC184 (complemented) strains were performed in the M9 supplemented with ornithine 0.7 mM or arginine 0.7 mM, under the same conditions. For the standard cultivation method, bacteria were inoculated in 100 ml media in flasks with M9-minimal medium, M9 supplemented with putrescine, spermidine, ornithine, or arginine with continuous shaking at 37 °C. At each time point (30 min intervals), OD_600_ values were checked out.

### Ultra-high performance liquid chromatography (UHPLC)

Intracellular concentrations of putrescine in the strains; WT-ST117, Δ*potE*-ST117 and Δ*speB/C*-ST117 were estimated by UHPLC. Samples were harvested at five different time points during growth in M9 and M9 supplemented with putrescine; T0- initial lag-phase (OD_600_ = 0.05), T1- start of the logarithmic phase (OD_600_ = 0.5), T2- mid-logarithmic phase (OD_600_ = 1), T3- start of the stationary phase (OD_600_ = 1.8–2), and T4- late stationary phase (OD_600_ = 3–4). The samples were centrifuged and washed with sterile PBS and immediately frozen and stored at − 20 °C.

For putrescine determination, the frozen cellular pellets were resuspended in 500 μL of 0.1 N HCl and boiled for 15 min. Then, a volume of 100 μL of the mixture was derivatized with diethyl ethoxymethylenemalonate (DEEMM) and separated by UHPLC following the protocol described in Redruello et al. [41]. Data were acquired and analyzed using Empower 2 software (Waters). All the chemicals used were of the highest available purity. The water of Milli-Q quality (Millipore) was used in all solutions. HPLC-grade acetonitrile (VWR) and methanol (Merck) were used as pure solvents. The putrescine concentration in total cellular homogenates is expressed as mg/L.

### Induction of membrane stress caused by SDS

The WT-ST117, the mutant Δ*potE*-ST117, and the complemented mutant Δ*potE*/pACYC184-ST117 strains were grown overnight in LB at 37 °C, 200 rpm, adjusted to an OD_600_ = 1, and 10-fold dilutions were prepared. 100 μl of each dilution were cultured on LB agar plates and LB agar plates supplemented with 0.01% (*w*/*v*) and 0.1% (w/v) SDS (Sodium dodecyl sulfate) (Thermo Scientific). CFU counts were assessed after 16–18 h of incubation at 37 °C. The experiment was performed in triplicate.

### RNA extraction and qPCR

To analyze the gene expression level of the genes *speF* and *speE* in the mutant ∆*speB/C*-STS117, bacteria were harvested at three different time points during growth in M9; start of the logarithmic phase, mid-logarithmic phase and stationary phase (OD_600_ = 0.5, OD_600_ = 1 and OD_600_ = 2, respectively). RNA was isolated from the cultures (1.5 ml) by mechanical disruption with the FastPrep (Bio101; Qbiogene) and RNeasy mini kit (Quiagen). Quantity and quality of total RNA were determined with the NanoDrop 1000 spectrophotometer (Thermo Scientific) and on a 1.5% (w/v) agarose gel. To remove chromosomal DNA, RNA samples were treated with RNase free DNase I kit (Thermo Scientific). The cDNAs were synthesized using the GoScript reverse transcriptase kit (Promega). The qPCR was done using the Maxima SYBR Green/Rox qPCR Master Mix and gene-specific oligonucleotides, which are described in the (Additional file 1: Table S3), in a LightCycler 96 (Roche). Data were normalized against the reference gene 16S rRNA [42] and normalization was determined based on the established mathematical model [43]. The experiment was performed in triplicate.

### Statistical analysis

Statistical analysis was performed using the GraphPad Prism (GraphPad Software) version 5.0. Differences between the mean growth rate of strains and between the amounts (mg/L) of putrescine in different strains were evaluated by students t-test. A *P*-value of < 0.05 was considered significant.

## Additional files

Additional file 1: **Figure S1**-**S5.** and **Tables S1-S3.** Description of Data: The additional file contains information on the genomic organization of *potE* in *E. coli* WT-ST117 and *E. coli* MG1655, growth phenotypes of polyamine biosynthesis mutants, growth performance of the mutants under stress conditions, expression levels of polyamine biosynthesis genes, correlation between CFU counting and OD values, antimicrobial phenotype results, and oligonucleotide sequences for PCR-based amplification. (PDF 614 kb)

## Abbreviations

Amp: Ampicillin
APEC: Avian pathogenic *Escherichia coli*
ATCC: American type culture collection
BLAST: Basic local alignment search tool
CFU: Colony formation unit
CGE: Center for Genomic Epidemiology
Chl: Chloramphenicol
ENA: European nucleotide archive
Gen: Gentamicin resistant
HPLC: High performance liquid chromatography
LB: Luria Bertani
M9: Minimal medium
MAUVE: Multiple genome alignment
OD: Optical density
PAI: Pathogenicity islands
PBS: Phosphate-buffered saline
PEG: Promoter extraction
qPCR: quantitative PCR
ROS: Reactive oxygen species
rpm: revolution per minute
SDS: Sodium dodecyl sulphate
Tet: Tetracycline
Tmp: Trimethoprim
UHPLC: Ultra-high performance liquid chromatography
VWR: Van waters and Rogers
*w*/*v*: weight per volume
WGS: Whole genome sequencing
WT: Wild type
