# Supplementary material for: Putrescine biosynthesis and export genes are essential for normal growth of avian pathogenic Escherichia coli
Source: BMC Microbiol. 2018 Dec 27;18:226. doi: 10.1186/s12866-018-1355-9 (PMC6307189; doi:10.1186/s12866-018-1355-9)
Supplement: Supplementary file 1 — Figure S1-S5. and Tables S1-S3. Description of Data: The additional file contains information on the genomic organization of potE in E. coli WT-ST117 and E. coli MG1655, growth phenotypes of polyamine biosynthesis mutants, growth performance of the mutants under stress conditions, expression levels of polyamine biosynthesis genes, correlation between CFU counting and OD values, antimicrobial phenotype results, and oligonucleotide sequences for PCR-based amplification. (PDF 614 kb) [file 12866_2018_1355_MOESM1_ESM.pdf]

Supplemental material

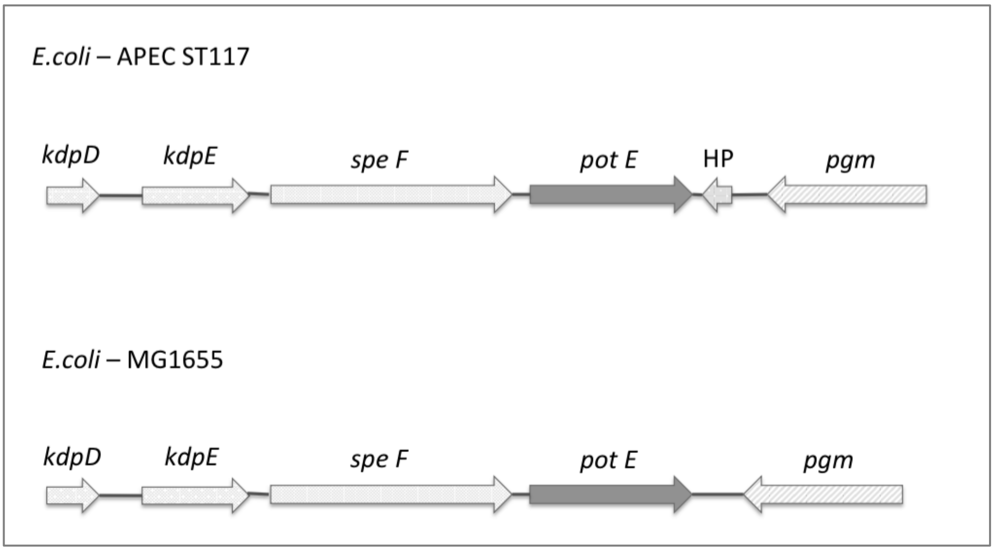

Fig S1 Schematic showing the genomic organization of *potE* gene in *E.coli* WT-ST117 and *E.coli* MG1655 based on WGS.

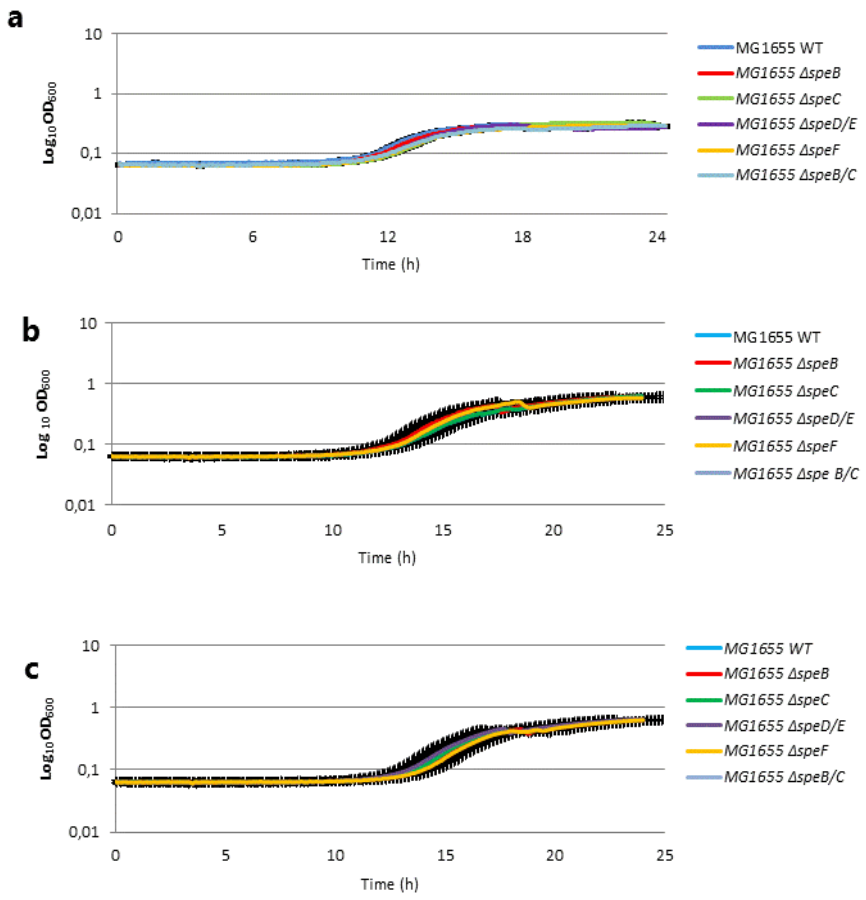

Fig S2A Growth phenotypes of polyamine biosynthesis mutants of *E.coli*-MG1655 in a) M9-minimal medium; b) M9-minimal medium supplemented with putrescine; c) M9-minimal medium supplemented with spermidine

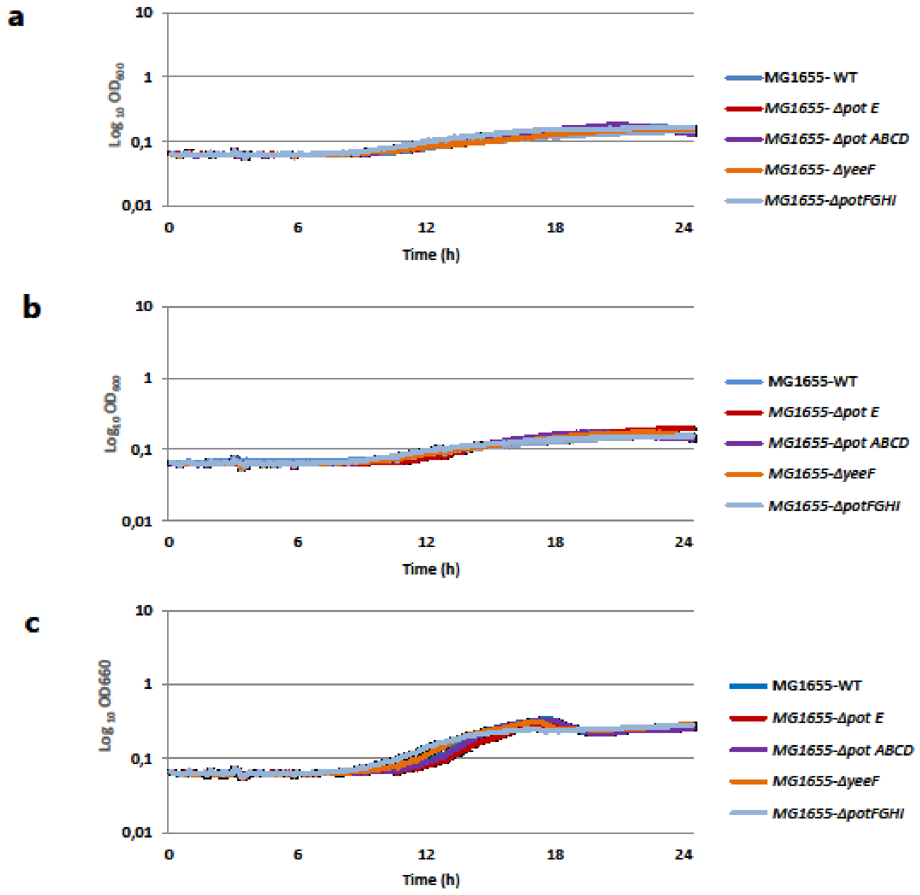

Fig S2B Growth phenotypes of polyamine transport mutants of *E.coli*-MG1655 in a) M9-minimal medium; b) M9-minimal medium supplemented with putrescine; c) M9-minimal medium supplemented with spermidine.

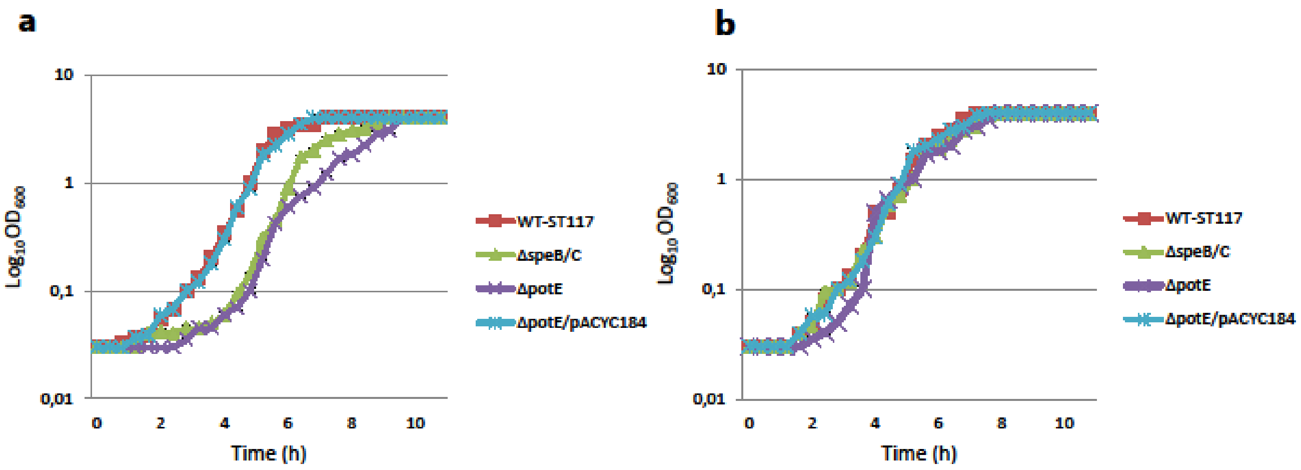

Fig S3 Growth curves determined by the standard cultivation method in a) M9-minimal medium; b) M9-minimal medium supplemented with putrescine.

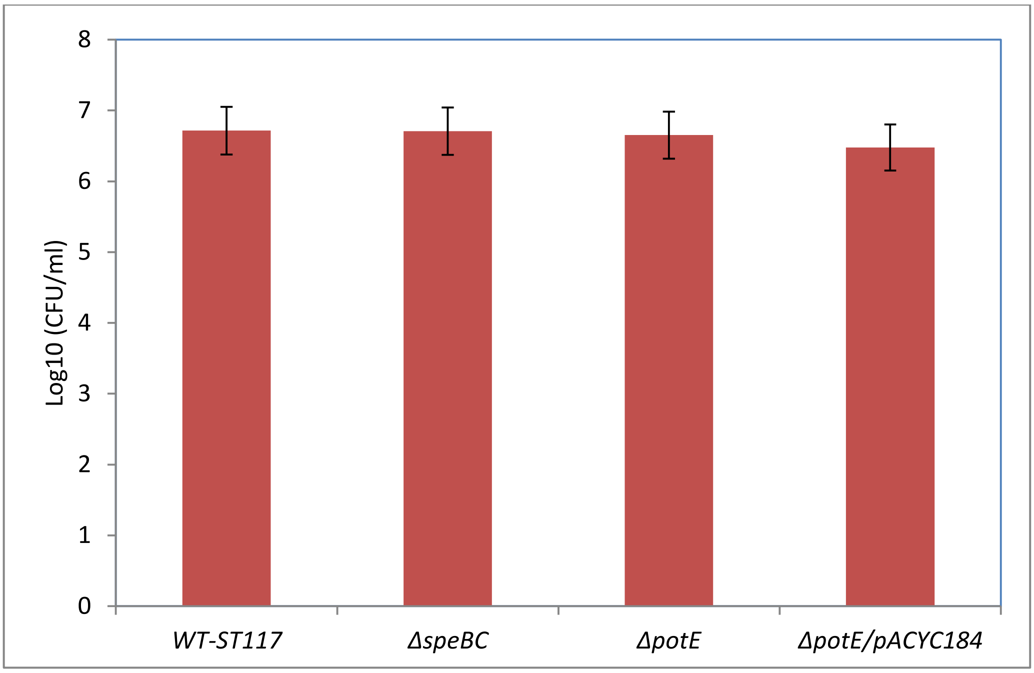

Fig S4 Growth of WT-ST117, Δ*potE*-ST117, and Δ*potE*/pACYC184 strains in the presence of 0.01% (w/v) SDS.

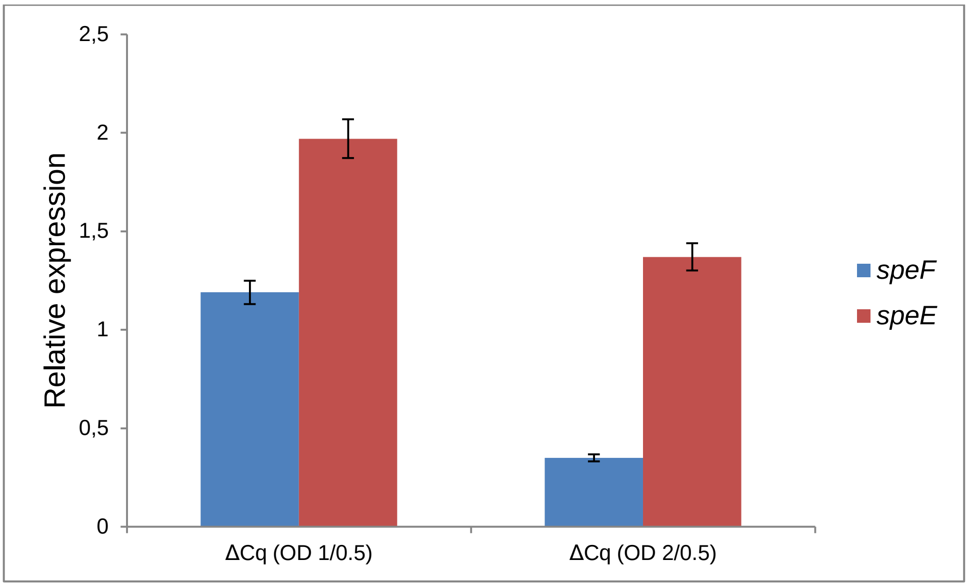

Fig S5 Expression levels of *speF* and *speE* genes in the mutant Δ*speB/C*-STS117 during growth in minimal medium.

Table S1 Correlation between CFU counting and OD_{600} values of WT, *ΔspeB/C*- ST117 and *ΔpotE*-ST117 grown in M9 medium supplemented with putrescine

	**WT-ST117**		***ΔspeB/C*- ST117**		***ΔpotE*- ST117**	
**time**	**CFU/ml**	**OD_{600}**	**CFU/ml**	**OD_{600}**	**CFU/ml**	**OD_{600}**
T0	4×10^{6}	0,064	$3{,}6 \times 10^{6}$	0,05	3×10^{6}	0,05
T1	$9{,}6 \times 10^{7}$	0,513	$8{,}9 \times 10^{7}$	0,55	$2{,}8 \times 10^{6}$	0,45
T2	$1{,}2 \times 10^{8}$	1,15	$1{,}6 \times 10^{8}$	1,42	$1{,}4 \times 10^{8}$	1
T3	$1{,}5 \times 10^{8}$	2,26	$7{,}8 \times 10^{7}$	1,9	$1{,}6 \times 10^{8}$	2
T4	6×10^{8}	3,15	9×10^{8}	2,8	$2{,}5 \times 10^{8}$	2,75

Table S2 Antimicrobial phenotypes of WT-ST117 strain detected by the disk-diffusion method.

Antimicrobial	mm	profile
Ampicillin	0	Resistant
Kanamycin	18	Susceptible
Chloramphenicol	22	Susceptible
Gentamicin	19	Susceptible
Trimethoprim	30	Susceptible

Table S3 Oligonucleotide sequences for PCR-based amplification.

Gene	Sequence	Primer application
*spe*B	fwd:$^{5'}$GTGATTTCCGACTGATCGTATGCCGGAGCCACTTCCA CTA GTGTAGGCTGGAGCTGCTTC$^{3'}$<br>rev:$^{5'}$AAGGGTTTTTTTATATCGACTTTGTAATAGGAGTCCAT CCCATATGAATATCCTCCTTAG$^{3'}$	recombination
*spe*B	fwd:$^{5'}$GTCAGGTAAACCGGCATATCACC$^{3'}$<br>rev:$^{5'}$GACTGGGTGATTACTGGCGTGC$^{3'}$	proof of insertion
*spe*C	fwd: $^{5'}$ACACAGACG GTTAGCCACT AATTACGCAAAGAAAAACGG GGTGTAGGCTGGAGCTGCTTC$^{3'}$<br>rev:$^{5'}$AGGGTTTTCCACCTTGTCCGGTATTCTTACTTCCCCGA AACATATGAATATCCTCCTTAG$^{3'}$	recombination
*spe*C	fwd: $^{5'}$TTACTTCAACACATAACCGTACAAC$^{3'}$<br>rev: $^{5'}$CAGCCTGTCAGTATGAAGAGAATTT$^{3'}$	proof of insertion
*spe*B/C	rev_up: $^{5'}$CCAAGGTTGAAACGAAAGCGCAAAACCCGTTTC$^{3'}$<br>rev_down: $^{5'}$TTGACGGAGGGCTTTAAAAAAACGGGTCACCTT CTG $^{3'}$<br>speC_fwd: $^{5'}$CACCACCAGTAAAATACCAATCC$^{3'}$<br>speC_rev: $^{5'}$GTTTGCCAGCAGCTTCAT$^{3'}$<br>dfrA14_fwd: $^{5'}$ACGGGTTTTGCGCTTTCGTTTCAACCTTGGTGT TTGG^{3}dfrA14_rev: $^{5'}$ GTGACCCGTTTTTTTAAAGCCCTCCGTCAATTTTATTACC$^{3'}$	Recombination by Infusion® method + proof of insertion
*spe*D/E	fwd: $^{5'}$TTCGCTGGCGATGAAATGGAAGAGGGGATGAACTACTAC G GTGTAGGCTGGAGCTGCTTC$^{3'}$<br>rev:$^{5'}$TTTTACGGGTGTTAACAAAGGAGGTATCAACCCATGG CCGCATATGAATATCCTCCTTAG$^{3'}$	recombination
*spe*D/E	fwd: $^{5'}$AAAGAAAACCTGGCCTTGCTT$^{3'}$<br>rev: $^{5'}$AACGCTACACGACCAGTTTGGGCA$^{3'}$<br>fwd: $^{5'}$GAAGATACCGCCAGGATTCA$^{3'}$<br>rev: $^{5'}$TGATCGACGATGGTGTCAAT$^{3'}$	proof of insertion/ gene expression assay
*spe*F	fwd: $^{5'}$TTTACGGCTG AACATACCGC ATTTGCGAATGCCCATGCC GTGTAGGCTGGAGCTGCTTC$^{3'}$<br>rev:$^{5'}$TTCTTCCGCGCACTGGTCGATTATGTCAATCACATATG AATATCCTCCTTAG$^{3'}$	recombination
